# Supplementary material for: Transthyretin Cardiac Amyloidosis: A Cardio-Orthopedic Disease
Source: Biomedicines. 2022 Dec 12;10(12):3226. doi: 10.3390/biomedicines10123226 (PMC9775219; doi:10.3390/biomedicines10123226)
Supplement: Supplementary file 1 [file biomedicines-10-03226-s001.zip › biomedicines-2043462-supplementary.pdf]

Table S1. Summary of key data from literature concerning the ATTR prevalence in some orthopaedic pathologies and the prevalence of amyloid deposits on surgical biopsies. Case reports are excluded.

Orthopedic Pathology	Authors	Subjects N°	Mean age(y)	Amyloid deposit	Key findings
**CTS**	Maurer et all [17]	189 ATTRwt<br>91 ATTRv	66	ND	History of carpal tunnel release was present in 33,3% of ATTRwt patients and in 29.1 ATTRv patients
	Nakagawa et al [18]	31	69.8	ND	CTS was the most common initial symptom in 17 (55%) patients with ATTRwt, followed by HF symptoms (n14, 45%)
	Sekijima et al [31]	51 ATTRwt	71.8	FTS	34 of 100 patients with idiopathic CTS had TTR amyloid deposits versus 7 of 32 autopsy controls
	Aus dem Siepem et al. [19]	253 ATTRwt<br>136 ATTRv<br>77 cATTRv	63.7	ND	Latency between CT surgery and diagnosis of systemic amyloidosis was significantly longer in ATTRwt compared to ATTRv (117±179 months vs 66±73 months; *p*=0.02)
	Sperry et al. [20]	98 patients ongoing CT surgery	68.5	TST	10 of 98 patients undergoing CT surgery had an amyloid positive biopsy (7 TTR, 2 light chain
	Fosbøl et al. [21]	56032 patients ongoing CT surgery	67.9	ND	CTS was associated with a higher cumulative incidence of amyloidosis and HF compared with matched control subjects. Median time from CTS surgery to amyloidosis diagnosis was 3.1.
	Vianello et al. [22]	53 male with bilateral CTS	72		Inexplicable left ventricular hypertrophy was found in 11% of patients with 2 patients that received a diagnosis of ATTRwt
	Milandri et al. [23]	166 ATTRv,<br>107 ATTRwt,<br>196 AL,<br>69 cTTRv	61.5	ND	CTS prevalence was highest in ATTR patients with CA (20.3% vs. 4.1%) than in the general population, while it was comparable to the general population whiteout CA and in AL pts
	Porcari et al. [25]	130 patients undergoing CT surgeries	81	ND	At the time of CT surgery, Unexplained LVH was found in more than 30% of patients with LVH, and 24% of them showed echocardiographic features suggesting an underdiagnosed CA.
	Ladefoged et al. [26]	120 patients with CTS surgery	74.5	ND	Among the study population the suspicion of ATTR was raised in 67 (55.8%) patients, and 10 (8.3%) received a diagnosis of ATTRwt.
**BBTR**	Geller et al. [29]	111 ATTRwt	74,7	ND	33.3% of 111 patients with ATTRwt cardiomyopathy had ruptured distal biceps tendon on exam
	Cappelli et al. [30]	168 ATTR-CA<br>81 HCM/AFD<br>93 controls<br>18 patients	77.9 56.9<br>76.8	ND	BBTR was present in 44% of ATTR-CA patients, 8% of controls and 1% in HCM/AFD.wt
**LSS**	Aus dem Siepem et al. [19]	253 ATTRwt<br>136 ATTRv<br>77 cATTRv	63.7	ND	36 patients (14%) with ATTR and 7 patients (5%) with ATTRv had a history of symptomatic LSS. A history of both conditions, was present in 32 patients with ATTRwt (12%) and 3 patients with ATTRv (2%)
	Sekijima et al [31] Wininger et al [35]	51 ATTRwt<br>157 biopsies	71.8<br>>70	ND<br>LF	At the time of diagnosis among the study population the presence of LSS was 22% TTR positive biopsies were identified in 64 of these patients and 0 had AL positive biopsies.
	Yanagisawa et al. [36]	56 patients (95 biopsies)	70.7	LF	All 95 LF specimens resected from LSS patients contained amyloid deposits; 43 contained TTR amyloid
	George et al. [37]	178 LF specimens	58.1	LF	24/ 178 patients (13.5%) were found to have ATTRwt in the LF. Patients with ATTRwt had thicker LF compared to patients without ATTRwt
	Eldhagen et al. [38]	250 patients ongoing LSS surgery	67.8	LF	Amyloid was identified in 221 specimens, 42 of whom of TTRwt. None of them have symptoms and sign of CA
	Godara et al. [39]	324 patients ongoing LSS surgery	66	LF	Amyloid was identified in 221 specimens, 43 of whom of TTRwt. 4 patients (11%) showed bone scan scintigraphy imaging suggestive for an underdiagnosed CA ATTR CA,
**Hip and Knee OA**	Egan et al. [40]	18 patients	68	Synovium	Amyloid (not typed) deposits were identified in 10/18 synovial specimens
	Mitrovic et al. [41]	53 knees & 26 femoral biopsies	NA	Synovium	Positive Congo red staining and thioflavin T fluorescence were found in 58.5% and 29% of the cases, in the knee and hip joint respectively
	Ladefoged et al. [42]	30 knees biopsies	71,2	Synovium	Knee joints from 28 of the 30 autopsy specimens contained amyloid deposits (93%).
	Takanashi et al. [43] Wininger et al [35]	232<br>382 biopsies	73<br>>70	Synovium<br>Synovium	21 of 232 OA patients (9%) had TTR amyloid deposits<br>97 biopsies positive fot ATTR 1 positive for AL
	Rubin et al. [44]	172 ATTR-CA<br>141 AL-CA	72.9 60	ND	THA and TKA significantly more common among ATTR patients with CA (THA: RR 5.61, 95% CI 2.25-4.64; TKA: RR 3.32, 95% CI 2.25-4.64)
**Trigger Finger**	Cordiner-Lawrie et al. [46]	47 patients	45	Tendon sheath	Amyloid deposits were found in the biopsies of 11/47(23%) patients (5 women and 6 men). Amyloid deposit were non characterized
	Hara et al. [48]	20	65.3	Synovium Tendon sheath	the presence of TTR amyloid deposition in the tenosynovium or flexor tendon biopsies was present in 13 (65%) patients.
	Sood et al. [49]	126,788 patients undergoing TR/CTR		Synovium Tendon sheath	Amyloidosis was diagnosed in 52 of 26,757 patients undergoing TR alone at a median of 4.7 years after surgery and in 50 of 8,647 patients undergoing both TR and CTR at a median of 3.1 years after surgery
**Rotator cuff disease**	Cardinal et al. [53]	19 patients on chronic HD	55	ND	The mean rotator cuff thickness on US from patients with dialysis-related amyloidosis was significantly greater (7 mm) than that in the control group (5 mm;p < .0001).
	Sommer et al. [54]	12 patients on chronic HD	59.5	ND	US findings suggestive for Amyloidosis shoulder involvements were observed in all patient examined
	Rubin et al. [44]	!72 ATTR-CA<br>141 AL-CA	72.9 60	ND	7 (5%) and 17 (9.9%) patients underwent rotator cuff repair
	Sueyoshi et al. [56]	111 patients specimens	65	FTS, LF, RCT	47 of 111 specimens contained amyloid deposits of whom 39 of TTRwt<br>8/21 (38%) specimen from RCT contained amyloid (5 TTRwt)

CTS carpal tunnel syndrome, HF heart failure, CA cardiac amyloidosis, HF heart Failure, cATTRv ATTRv carriers ; AL : light Chain Amyloidosis ,LVH left ventricular hypertrophy, FTS flexor tenosynovium, BBTR Brachial biceps tendon rupture, HCM hypertrophic cardiomyopathy, AFD Anderson Fabry disease, LSS lumbar spinal stenosis, LF ligamentum flavum, OA osteoarthritis, THA total hip arthroplasty, TKA total knee arthroplasty, TR trigger release, CTR carpal tunnel release, HD haemodialysis , US ultrasonography.
